# An association between time-varying serum albumin level and the mortality rate in maintenance haemodialysis patients: a five-year clinical cohort study

**DOI:** 10.1186/s12882-016-0332-5

**Published:** 2016-08-20

**Authors:** Jin-Bor Chen, Ben-Chung Cheng, Cheng-Hong Yang, Moi-Sin Hua

**Affiliations:** 1 Division of Nephrology, Department of Internal Medicine, Kaohsiung Chang Gung Memorial Hospital and Chang Gung University College of Medicine, 123 Ta Pei Rd, Niao Song District, Kaohsiung, Taiwan; 2 Department of Electronic Engineering, National Kaohsiung University of Applied Sciences, Kaohsiung, Taiwan

**Keywords:** Albumin, Mortality, Haemodialysis

## Abstract

**Background:**

Until now, no long-term studies relating serum albumin level to mortality rate in prevalent haemodialysis (HD) patients have been conducted. We aimed to examine the association between serum albumin level and mortality over a 5-year period.

**Methods:**

This study included 781 patients who received maintenance HD in a large, hospital-facilitated HD centre. Five-year medical records (2009–2013) were retrospectively reviewed, and the cut-off level for serum albumin level was set at 3.5 g/dL. The analysed albumin levels were expressed as time-averaged levels (first 24-month data) and albumin target reach rate over the first 2-year interval. Univariate and multivariate Cox proportional hazard regression models were used to examine the hazard function of the all-cause and cardiovascular mortality of the study participants in the subsequent 3-year period (2011–2013).

**Results:**

Compared to those with a 100 % albumin reach rate (3.5 g/dL), the participants with 75– < 100, 50– < 75, and 1– < 50 % albumin reach rates exhibited significantly increased risk for all-cause mortality (HR 1.72, 95 % CI 1.19–2.47; HR 3.14, 95 % CI 1.91–5.16; HR 3.66, 95 % CI 2.18–6.16, respectively). A similar trend for all-cause mortality was demonstrated in participants with time-averaged albumin levels <4 g/dL (HR 1.57, 95 % CI 1.00–2.46 for 3.5–4.0 g/dL; HR 3.66, 95 % CI 2.11–6.32 for <3.5 g/dL). Compared to a 100 % albumin reach rate, the 50– < 75 and 1– < 50 % groups (HR 4.28, 95 % CI 1.82–10.01; HR 3.23, 95 % CI 1.22–8.54 respectively) showed significantly higher cardiovascular mortality rates. Similarly, participants with a time-averaged serum albumin level <3.5 g/dL exhibited a higher risk for cardiovascular mortality (HR 3.24, 95 % CI: 1.23–8.56).

**Conclusions:**

This long-term study demonstrated that higher reach rates of serum albumin levels and higher time-averaged serum albumin levels are associated with a lower mortality rate in patients undergoing maintenance HD.

## Background

Serum albumin is commonly used as a proxy for nutritional status, as well as a marker of inflammation. A low serum albumin concentration is not only indicative of protein energy wasting in dialysis patients, but it is also a powerful predictor of the mortality risk in this population [1–4]. In addition, there is accumulating evidence that the factors causing the low albumin levels, rather than hypoalbuminaemia per se, may be associated with high mortality and morbidity in dialysis patients [5, 6]. Several clinical conditions are associated with low serum albumin levels in dialysis patients, including infectious and inflammatory diseases [7, 8], fluid overload, inadequate dialysis [9], severe co-morbidity [10], and taste change [11]. Thus, regular monitoring of serum albumin levels is useful for predicting outcomes in dialysis patients.

In previous studies, the association between the serum albumin level and mortality in dialysis patients was based either on a baseline serum albumin level or on a mean level for the study period. One study that used time-dependent 2-year longitudinal change analysis noted that a drop in serum albumin levels during the first 6 months was associated with an increase in all-cause and cardiovascular mortality in the subsequent months [2]. However, current studies are limited by their short-term nature and relatively small sample sizes.

In the present 5-year study, we investigated the percentage of each patient’s serum albumin levels that reached a pre-defined target (3.5 g/dL) during the study period. This percentage was termed the serum albumin reach rate in this study. We aimed to examine the association between time-averaged albumin and serum albumin reach rate in the first 2-year period and the risk of mortality in the subsequent 3-year period in prevalent haemodialysis (HD) patients.

## Methods

### Setting

Patients who received regular outpatient HD (3 times per week) at Kaohsiung Chang Gung Memorial Hospital in Taiwan were included in the study. The patients were tracked from January 1, 2009 to December 31, 2013.

### Patients

The records of 909 HD patients were reviewed, and 128 patients were excluded owing to incomplete demographic and laboratory data in the study period. Finally, 781 patients were considered eligible for inclusion in the survival analysis. Patient number of high flux HD were 672, high efficiency HD 109. The types of vascular access were arteriovenous fistula 731, Gore-Tex access 50. The underlying kidney diseases were renal parenchymal diseases (*n* = 156), systemic diseases (*n* = 392), obstructive nephropathy (*n* = 6), hereditary disease (*n* = 4), unknown cause (*n* = 221), poisoning (*n* = 2). During the study period, 182 patients died and 599 patients survived.

### Study design

The study was a 5-year retrospective observational study. Serial haemogram results and biochemical data from the study period were collected and were analysed retrospectively. Baseline values were measured in 2009, including haemoglobin (Hb), albumin, blood urea nitrogen (BUN), creatinine (Cr), potassium (K), corrected serum calcium (Ca), phosphate (P), urea reduction ratio (URR), intact parathyroid hormone, ferritin, Kt/V urea (Daugirdas method) [12], and cardiothoracic (CT) ratio. Corrected serum calcium was calculated by using the following equation: measured total Ca (mg/dL) + 0.8 (4.0 − serum albumin [g/dL]). All blood samples were analysed using commercial kits and an autoanalyzer (Hitachi 7600–210, Hitachi Ltd., Tokyo, Japan). Albumin was measured using the bromocresol green method; the normal range was 3.5–5.2 g/dL. Chest radiography for the measurement of the CT ratio was performed after HD. All patients received HD with dialyzers that had an effective surface area of >2.0 m^2^.

To examine the association between serum albumin levels and mortality, we used time-averaged albumin level and serum albumin reach rate to 3.5 g/dL in the first 2 years (2009–2010) as the predictor variables and all-cause mortality and cardiovascular mortality in the subsequent 3 years (2011–2013) as outcome variables. The time-averaged albumin level was the average of the 2-year (24 months) serum albumin level. Twenty-four or less (censored) measurements of serum albumin levels were observed over the first 2 years for each patient. We defined serum albumin reach rate in the first 2 years as the percentage of albumin levels that reached 3.5 g/dL and the percentage of albumin levels that were ≥3.5 g/dL. First, we stratified the albumin reach rate into 5 groups: 100 % (all 24 monthly serum albumin levels reached 3.5 g/dL), 75– < 100, 50– < 75, 1– < 50, and 0 % (none of the 24 monthly serum albumin levels reached 3.5 g/dL). Second, we divided the participants into 2 groups according to the percentage of monthly serum albumin level recordings that were ≥3.5 g/dL over the first 2-year follow-up period: higher rate group (≥75 % serum albumin levels were ≥3.5 g/dL) and lower rate group (<75 % of serum albumin levels were ≥3.5 g/dL).

The protocol for the study was approved by the Committee on Human Research at Kaohsiung Chang Gung Memorial Hospital (101-1595B) for data review and was conducted in accordance with the Declaration of Helsinki. Informed consent was not required from the study patients, according to the retrospective data review regulations of the Committee on Human Research at Kaohsiung Chang Gung Memorial Hospital.

### Outcome measures

The outcome measures included the associations between serum albumin reach rates (defined as the percentage of monthly serum albumin level recordings that were ≥3.5 g/dL) and time-averaged albumin levels in the first 2-year period with all-cause and cardiovascular mortality in the subsequent 3-year period.

### Statistical analysis

All statistical analyses were carried out by using STATA (Version 11.1). For descriptive statistics, all variables were calculated as means ± SD (standard deviation), median with interquartile ranges, and frequency and proportion of patients. For categorical variables, the differences between groups were estimated using the *χ*^2^ test or Fisher’s exact test as appropriate. For continuous variables, the differences between groups were estimated with an independent two-sample *t*-test. In addition to descriptive statistics, both univariate and multivariate linear regression were used to analyse the association between serum albumin and baseline relevant variables. The univariate and multivariate Cox proportional hazard regression model was used to estimate the hazard function of all-cause and cardiovascular mortality for study participants, and the hazard ratios (HR), 95 % confidence intervals (95 % CI), and *p*-values were computed. The stratified analysis for higher albumin rate and lower albumin rate participants was performed by using the Cox regression model. In all analyses, a *p*-value < 0.05 is considered statistically significant.

## Results

A total of 781 patients were enrolled for the analysis. Among them, 689 patients had higher albumin reach rates and 92 patients had lower albumin reach rates over the 5-year period. Older age and higher prevalence of diabetes were identified in the patients with lower albumin reach rates. Patients with higher albumin reach rates exhibited higher survival rates than those with lower albumin reach rates. In terms of haemogram and biophysical parameters, patients with lower albumin reach rates exhibited significantly lower values for albumin, BUN, Cr, K, and P and higher levels of Ca, ferritin, and CT ratio than those with higher albumin reach rates (Table 1).

**Table 1:** Baseline characteristics and clinical features of the study population with different albumin status (*n* = 781)

Variables	Albumin rate reached 3.5 (g/dL)				*P*
	Lower rate (*n* = 92)		Higher rate (*n* = 689)		
	Mean	SD	Mean	SD	
Dialysis vintage (years)	7.29	4.94	7.81	4.98	0.346
5-year mortality (*n*, %)					<0.001
Survival	40	43.48	559	81.13	
Cardiovascular	18	19.57	37	5.37	
Other cause	34	36.96	93	13.50	
Age (years)	66.60	11.48	58.32	11.88	<0.001
Gender (*n*, %)					0.066
Male	33	35.87	317	46.01	
Female	59	64.13	372	53.99	
Diabetes Mellitus (*n*, %)					<0.001
No	56	60.87	540	78.37	
Yes	36	39.13	149	21.63	
Year of death					–
Death before 2010	18	19.57	19	2.76	
Death after 2010	34	36.96	111	16.11	
Blood analysis					
Hb (g/dL)	10.37	1.68	10.76	1.23	0.006
Albumin (g/dL)	3.43	0.30	3.95	0.27	<0.001
BUN (mg/dL)	62.67	18.74	69.26	16.12	<0.001
Cr (mg/dL)	8.75	2.22	10.89	2.19	<0.001
K (meq/L)	4.73	0.77	4.99	0.71	<0.001
Ca (mg/dL)	9.54	0.81	9.34	0.85	0.030
P (mg/dL)	4.54	1.52	4.89	1.38	0.023
iPTH (pg/mL)	163.45	(60–356)	216.10	(76–510)	0.141
Ferritin (ng/mL)	443.25	(280–714)	396.10	(231–595)	0.022
Kt/V urea	1.69	0.34	1.74	0.78	0.540
URR	0.75	0.07	0.75	0.07	0.942
Cardiothoracic ratio	0.54	0.08	0.50	0.07	<0.001

*P*-value for categorical variable was estimated by *χ* ^2^ test and continuous variables were estimated by two-sample *t*-test

*Abbreviations*: *URR* urea reduction ratio

The univariate and multivariate regression analysis between baseline serum albumin level and relevant variables is shown in Table 2. In the univariate regression analysis, baseline albumin level had a statistically significant inverse association with age, female sex, diabetes, Ca level, and CT ratio. For female sex participants, the predicted serum albumin levels would be 0.11 g/dL lower than for males. Likewise, the predicted serum albumin levels of diatebes participants would be 0.08 g/dL lower than non-diabetes participants. Moreover, baseline albumin level had a statistically significant positive association with Hb, BUN, Cr, K, and P levels. In the multivariate regression analysis, baseline albumin level had a statistically significant inverse association with age, and a positive association with Hb, Cr, K, ferritin levels, and URR.

**Table 2:** Univariate and multivariate regression analysis between baseline serum albumin levels and relevant variables

Variable	Beta^a^	*P* ^a^	Beta^b^	*P* ^b^
Dialysis vintage (years)	0.01	0.709	−0.03	0.41
Age (years)	−0.29	<0.001	−0.17	0.00
Gender (Female)	−0.11	0.002	0.02	0.61
Diabetes Mellitus	−0.08	0.021	0.01	0.87
Hb (g/dL)	0.24	<0.001	0.18	0.00
BUN (mg/dL)	0.20	<0.001	−0.02	0.57
Cr (mg/dL)	0.39	<0.001	0.29	0.00
K (meq/L)	0.22	<0.001	0.16	0.00
Ca (mg/dL)	−0.09	0.015	−0.03	0.34
P (mg/dL)	0.16	<0.001	0.05	0.19
iPTH (pg/mL)	0.01	0.738	−0.03	0.41
Ferritin (ng/mL)	−0.05	0.162	0.07	0.04
Kt/V urea	<0.01	0.946	0.00	0.91
URR	−0.02	0.551	0.08	0.04
Cardiothoracic ratio	−0.15	<0.001	−0.03	0.37

^a^Univariate regression analysis

^b^Multivariate regression analysis

Table 3 shows the distribution of the study population in different albumin categories (using 2-year serum albumin measurement) as well as by mortality based on the different serum albumin categories. The percentage of all-cause mortality and cardiovascular mortality over the 3-year period was significantly higher with lower time-averaged serum albumin level and with lower albumin reach rates over the first 2-year period.

**Table 3:** Different albumin categories using 2-year serum albumin measurement in the study population

Albumin group	Total		All-cause mortality			Cardiovascular mortality		
	*n*	%	*n*	%^a^	*P*	*n*	%^b^	*P*
Time-averaged serum albumin					<0.001*			<0.001*
≥4 g/dL	235	30.09	25	10.64		8	3.40	
3.5– < 4 g/dL	474	60.69	115	24.26		33	7.00	
<3.5 g/dL	72	9.22	42	58.33		14	19.44	
Albumin rate reached 3.5 (g/dL)					<0.001**			<0.001**
100 %	487	62.36	67	13.76		19	3.90	
75 to <100 %	202	25.86	63	31.19		18	8.91	
50 to <75 %	46	5.89	23	50.00		9	19.56	
1 to <50 %	38	4.87	24	63.16		7	18.42	
0	8	1.02	5	62.50		2	25.00	
Albumin rate reached 3.5 (g/dL)					<0.001*			<0.001*
Higher rate (75–100 %)	689	88.22	130	18.87		37	5.37	
Lower rate (0– < 75 %)	92	11.78	52	56.52		18	19.56	

*: *χ* ^2^ test

**: Fisher’s exact test

^a^(number of all-cause mortality/total number in each category) × 100 %

^b^(number of cardiovascular mortality/total number in each category) × 100 %

The Cox regression survival analysis shown in Table 4 demonstrates the associations between different albumin categories (using the first 2-year serum albumin measurement) and all-cause and cardiovascular mortality over the subsequent 3-year period. The 100 % albumin reach rate, higher reach rate, and time-averaged serum albumin ≥4 were set as the references. Compared to a 100 % albumin reach rate the 75– < 100, 50– < 75, and 1– < 50 % albumin reach rates exhibited significant increased risk in all-cause mortality according to the total adjusted analysis (HR 1.72, 95 % CI 1.19–2.47; HR 3.14, 95 % CI 1.91–5.16; and HR 3.66, 95 % CI 2.18–6.16, respectively) A similar trend in all-cause mortality was demonstrated with higher rate albumin reach (75–100 %) and higher time-averaged albumin levels. Compared to a 100 % albumin reach rate, 50– < 75 and 1– < 50 % reach rates were associated with significantly higher cardiovascular mortality (HR 4.28, 95 % CI 1.82–10.01 and HR 3.23, 95 % CI 1.22–8.54, respectively). Similarly, time-averaged serum albumin levels <3.5 g/dL conferred higher risk of cardiovascular mortality (HR 3.24, 95 % CI: 1.23–8.56) (Table 4).

**Table 4:** Associations between different albumin categories using 2-year serum albumin measurements with all-cause and cardiovascular mortality in the study population

Albumin category	All-cause mortality								
	Unadjusted			Adjusted			Adjusted		
				(demographic)					
	HR	95 % CI	*P*	HR	95 % CI	*P*	HR	95 % CI	*P*
Albumin rate reached 3.5 (g/dL)									
100 %	Reference			Reference			Reference		
75 to < 100 %	2.41	1.70–3.43	<0.001	2.01	1.41–2.87	<0.001	1.72	1.19–2.47	0.004
50 to < 75 %	5.82	3.61–9.37	<0.001	3.64	2.22–5.95	<0.001	3.14	1.91–5.16	<0.001
1 to < 50 %	7.20	4.43–11.69	<0.001	4.95	3.00–8.16	<0.001	3.66	2.18–6.16	<0.001
0	8.10	3.25–20.14	<0.001	4.80	1.90–12.15	0.001	2.54	0.96–6.76	0.062
Albumin rate reached 3.5 (g/dL)									
Higher rate (75–100 %)	Reference			Reference			Reference		
Lower rate (0– < 75 %)	4.45	3.22–6.14	<0.001	3.11	2.23–4.35	<0.001	2.55	1.79–3.63	<0.001
Time-averaged serum albumin									
≥4	Reference			Reference			Reference		
3.5 to < 4	2.38	1.54–3.68	<0.001	1.80	1.15–2.81	0.010	1.57	1.00–2.46	0.051
<3.5	8.71	5.28–14.37	<0.001	4.84	2.84–8.26	<0.001	3.66	2.11–6.32	<0.001
Albumin rate reached 3.5 (g/dL)									
100 %	Reference			Reference			Reference		
75 to < 100 %	2.32	1.18–4.54	0.015	1.99	1.01–3.93	0.046	1.73	0.86–3.48	0.124
50 to < 75 %	7.88	3.53–17.58	<0.001	4.78	2.09–10.96	<0.001	4.28	1.82–10.01	0.001
1 to < 50 %	7.75	3.23–18.62	<0.001	4.69	1.91–11.49	0.001	3.23	1.22–8.54	0.018
0	10.38	2.40–44.91	0.002	6.59	1.47–29.61	0.014	4.08	0.86–19.39	0.077
Albumin category	Cardiovascular mortality								
	Unadjusted			Adjusted			Adjusted		
				(demographic)					
	HR	95 % CI	*P*	HR	95 % CI	*P*	HR	95 % CI	*P*
Albumin rate reached 3.5 (g/dL)									
Higher rate (75–100 %)	Reference			Reference			Reference		
Lower rate (0– < 75 %)	5.88	3.31–10.45	<0.001	3.66	2.01–6.68	<0.001	2.94	1.58–5.47	0.001
Time-averaged serum albumin									
≥4	Reference			Reference			Reference		
3.5 to < 4	2.02	0.93–4.42	0.076	1.51	0.68–3.36	0.315	1.28	0.57–2.90	0.552
<3.5	8.94	3.74–21.37	<0.001	4.61	1.82–11.63	0.001	3.24	1.23–8.56	0.018

Univariate and multivariate Cox proportional hazard regression model

Adjusted (demographic), the adjusted covariates including dialysis vintage, age, sex, and diabetes mellitus

Adjusted, the adjusted covariates including dialysis vintage, age, sex, diabetes mellitus, and all laboratory blood analysis values

## Discussion

Serum albumin level is recognized as a strong predictor of mortality in HD patients [1–4]. However, serum albumin levels in routine blood tests are known to undergo dynamic changes. Therefore, a majority of clinical studies have used baseline levels, mean levels during a short-term period, or time-varying albumin levels to predict the risk of mortality in HD patients [1–4]. The present study investigated the association of 2-year serum albumin levels with mortality in a longitudinal 3-year period by utilizing the serum albumin reach rate for a pre-defined target level and the time-averaged albumin level. The cut-off serum albumin level was set at 3.5 g/dL, based on the lower normality limit in our laboratory test and the minimal requirement of the nutritional quality by the National Health Insurance Administration in Taiwan (http://sc-dr.tw/news/104/010603.htm) (http://sc-dr.tw/news/104/01/01060301.pdf). The study demonstrated that HD patients who reached the target serum albumin levels more frequently and who had higher time-averaged albumin levels over a 2-year period had a survival advantage in the subsequent 3-year period.

In this study, the baseline serum albumin level was found to be inversely associated with age, female sex, diabetes, Ca level, and CT ratio. Furthermore, the baseline serum albumin was found to be positively associated with, Hb, BUN, Cr, K, and P levels through univariate regression analysis. However, age was the only factor found to be inversely associated by multivariate regression analysis. A plausible explanation is that age is associated with a greater risk of disability and as well as limitation of oral intake. These conditions can result in protein malnutrition and a low albumin level.

Multivariate regression analysis showed that baseline serum albumin level was positively associated with Hb, Cr, K, ferritin levels, and URR. This is consistent with a previous study on a cohort of HD patients, which showed the association of 3-month average serum albumin levels with Hb and Cr levels [2]. It is reasonable to assume that patients with higher Hb levels may have greater activity levels and thus higher dietary intake. Furthermore, a higher serum Cr level potentially reflects a higher muscle mass and indicates a healthy nutritional status. It is also reasonable to assume that free access to dietary intake may result in a positive association of serum albumin level with serum K levels.

The adequacy of dialysis is critical for predicting morbidity and mortality among HD patients. One retrospective study of approximately 10,000 HD patients found that lower urea reduction ratios during dialysis were associated with increased risk of death; the risk was increased owing to inadequate nutrition [9]. Another study of 1600 HD patients concluded that, after reaching adequacy targets (Kt/V urea, URR), the main determinants of survival were nutrition status and age [13]. Our participants had adequate Kt/V urea scores, and a majority had adequate URR. Furthermore, a positive association was observed between URR and the baseline serum albumin level. Accordingly, our results are consistent with the previous findings that URR and serum albumin level may contribute to the risk of death in HD patients.

Previous studies have reported the association of serum albumin levels with mortality in dialysis patients [1–4, 7, 10]. However, most of the studies had short-term observational designs with durations between 1 and 2 years. Moreover, the outcome measures in prior studies were the baseline albumin level or the time-varying/average albumin level during the study period. In an earlier study with a follow-up of 1.25 years, baseline serum albumin level was found to be a strong predictor of death in chronic HD patients [1]. Another study with a 10-year observation period also showed that reduced baseline serum albumin level was an independent predictor of mortality in incident dialysis patients [10]. One study used trimonthly-varying serum albumin level and baseline albumin level to predict the mortality in HD patients over a 2-year follow-up period. The study demonstrated that the hazard ratios for cardiovascular death increase across decrements of baseline serum albumin levels; however, the hazard ratios of cardiovascular death did not differ with time-varying albumin levels <3.8 g/dL [2].

The present study is the first to utilize the serum albumin reach rate in prevalent HD patients. In addition, this study had a longer observational period than previous studies. We found that higher albumin reach rate and time-averaged serum albumin level over a 2-year period are associated with lower all-cause mortality over a 3-year follow-up period. A similar trend was also observed in association with cardiovascular mortality.

While most studies have investigated the association of serum albumin levels with all-cause mortality or cardiovascular mortality, one study conducted in Canadian HD patients found that the serum albumin levels were associated with septicaemia and hospitalization in patients with infectious diseases [7]. In the present study, we did not determine the exact cause of mortality; therefore, the weight contribution of septicaemia in all-cause mortality was not ascertained. Taken together, the results of the previous studies and that of our present study were consistent with regards to the association between serum albumin levels and mortality in dialysis patients, with either time-averaged albumin levels or albumin reach rate used as predictors.

Although the present study showed a significant association between low serum albumin levels and mortality in prevalent HD patients, chance findings cannot be completely excluded. Retrospectively selecting participants from one large HD centre may have influenced the results. Therefore, the association between the predictor variables and mortality may be overestimated. Furthermore, the present study did not investigate the contributions of inflammation, comorbidities, and nutritional status. Finally, we used albumin reach percentage in the first 2 years and time-averaged albumin level to predict mortality in the subsequent 3 years in our HD patients. However, there still exists a drawback for fully evaluation the exposure (albumin level) and mortality in our HD patients. A well-designed, prospective study is needed to overcome the drawback in retrospective study in the future.

## Conclusions

The present study indicates that a higher serum albumin level has a survival benefit in long-term HD patients. An obviously low albumin level should emphasize the need for initiating a management strategy and receiving appropriate medical intervention.

## Abbreviations

BUN, blood urea nitrogen; Ca, corrected serum calcium; CI, confidence interval; Cr, creatinine; CT ratio, cardiothoracic ratio; Hb, haemoglobin; HD, haemodialysis; HR, hazard ratio; K, potassium; P, phosphate; URR, urea reduction ratio

## References

[1] Iseki K, Kawazoe N, Fukiyama K (1993). Serum albumin is a strong predictor of death in chronic dialysis patients. Kidney Int.

[2] Kalantar-Zadeh K, Kilpatrick RD, Kuwae N, McAllister CJ, Alcom H, Kopple JD (2005). Revisiting mortality predictability of serum albumin in the dialysis population: time dependency, longitudinal changes and population-attributable fraction. Nephrol Dial Transplant.

[3] Amaral S, Hwang W, Fivush B, Neu A, Frankenfield D, Furth S (2008). Serum albumin level and risk for mortality and hospitalization in adolescents on hemodialysis. Clin J Am Soc Nephrol.

[4] de Mutsert R, Grootendorst DC, Indemans F, Boeschoten EW, Krediet RT, Dekker FW (2009). Association between serum albumin and mortality in dialysis patients is partly explained by inflammation, and not by malnutrition. J Ren Nutr.

[5] Yeun JY, Kaysen GA (1998). Factors influencing serum albumin in dialysis patients. Am J Kidney Dis.

[6] Don BR, Kaysen G (2004). Serum albumin: relationship to inflammation and nutrition. Semin Dial.

[7] Churchill DN, Taylor DW, Cook RJ, Laplante P, Barre P, Cartler P (1992). Canadian hemodialysis morbidity study. Am J Kidney Dis.

[8] Kaysen GA, Stevenson FT, Depner TA (1997). Determinants of albumin concentration in hemodialysis patients. Am J Kidney Dis.

[9] Owen WF, Lew NL, Liu Y, Lowrie EG, Lazarus JM (1993). The urea reduction ratio and serum albumin concentration as predictors of mortality in patients undergoing hemodialysis. N Engl J Med.

[10] Chan M, Kelly J, Batterham M, Tapsell L (2012). Malnutrition (subjective global assessment) scores and serum albumin levels, but not body mass index values, at initiation of dialysis are independent predictors of mortality: a 10-year clinical cohort study. J Ren Nutr.

[11] Steiber AL (1999). Clinical indicators associated with poor oral intake of patients with chronic renal failure. J Ren Nutr.

[12] Daugirdas JT (1989). The post: pre-dialysis plasma urea nitrogen ratio to estimate K.t/V and NPCR: mathematical modeling. Int J Artif Organs.

[13] Combe C, Chauveau P, Laville M, Fouque D, Azar R, Cano N (2001). Influence of nutritional factors and hemodialysis adequacy on the survival of 1,610 French patients. Am J Kidney Dis.

